# Supplementary material for: Association between complete blood-count-based inflammatory scores and hypertension in persons living with and without HIV in Zambia
Source: PLoS One. 2024 Nov 11;19(11):e0313484. doi: 10.1371/journal.pone.0313484 (PMC11554205; doi:10.1371/journal.pone.0313484)
Supplement: Attachment — Submitted filename: Response to Reviewers.docx [file pone.0313484.s003.docx]

Mulungushi University,

P.O Box 60009,

Livingstone.

1^st^ September, 2024.

The Editor and Reviewers,

PLOS ONE Journal.

Dear PLOS ONE Reviewers,

**RE: RESPONSE TO REVIEWERS COMMENTS**

Thank you so much for reviewing our manuscript and providing invaluable reviews. We appreciate your review comments which we believe have improved our manuscript's readability and interest.

We have taken time to correct our manuscript and provided a Track changes copy to reflect all changes in the manuscript. We have made substantial revisions in addition to what the reviewers requested including converting data with continuous variables from table 1 and 2 into figures to improve and enhance interest.

Below are our responses to the editor and reviewer comments.

**EDITOR COMMENTS**

When submitting your revision, we need you to address these additional requirements.

1. Please ensure that your manuscript meets PLOS ONE's style requirements, including those for file naming. The PLOS ONE style templates can be found at 
<https://journals.plos.org/plosone/s/file?id=wjVg/PLOSOne_formatting_sample_main_body.pdf> and <https://journals.plos.org/plosone/s/file?id=ba62/PLOSOne_formatting_sample_title_authors_affiliations.pdf>

**Response from authors:** Thank you. We have referred to the comments and have since adjusted the naming of the manuscript and supporting documents to PLOS ONE’s style requirements.

2. PLOS require an ORCID iD for the corresponding author in Editor Manager on paper submitted after December 6^th^ 2016. Please ensure that you have an ORCID iD and that it is validated in Editorial manager. To do this, go to ‘Update my information’ (in the upper left-hand corner of the main menu), and click on the Fetch/Validate link next to the ORCID field. This will take you to the ORCID site and allow you to create a new iD or authenticate a pre-existing iD in Editorial Manager. Please see the following video for instructions on linking an ORCID iD to your Editorial Manager account: <https://www.youtube.com/watch?v=_xcclfuvtxQ>

**Response from authors:** Thank you. We have managed to get an ORCID iD

3. Please include captions for your Supporting Information files at the end of your manuscript, and update any in-text citations to match accordingly. Please see our Supporting Information guidelines for more information:<http://journals.plos.org/plosone/s/supporting-information>.

**Response from authors:** Thank you, we have included the captions as per recommendation.

**RESPONSES TO REVIEWER COMMENTS**

**REVIEWER 1**
1. Define abbreviation in their first use and make sure that abbreviated forms are being used after definitions.

**Response from authors:** Thank you very much. We have made the revisions and defined all abbreviations upon first use, thereafter, we used the abbreviated form.

2. Add the clinical utility of your findings for a primary care physician?

**Response from authors:** Thank you so much. We have revised and included the clinical utility and implications of our findings in discussion section.

3. I found some typos and grammatical errors. A native review is warranted

**Response from authors:** Thank you so much for the comment. We have revised all the typos and grammatical errors were corrected as advised.

**REVIEWER 2**
1. The authors should provide a stronger emphasis on the clinical implications of their findings, which are currently not clearly articulated

**Response from author:** Thank you so much for the comment. We have clearly articulated and emphasized the clinical implications of our findings in the discussion section.

We want to thank the reviewers again for taking time to review and make suggestions that have improved our manuscript. We now hope it is acceptable for publication.

Yours,

Lackson Mwape

On behalf of the authors
